# Relationships between antenatal and postnatal care and post-partum modern contraceptive use: evidence from population surveys in Kenya and Zambia

**DOI:** 10.1186/1472-6963-13-6

**Published:** 2013-01-04

**Authors:** Mai Do, David Hotchkiss

**Affiliations:** 1 Department of Global Health Systems and Development, Tulane University School of Public Health and Tropical Medicine, 1440 Canal Street, Suite 2200, New Orleans, LA, 70112, USA

**Keywords:** Antenatal care, Postnatal care, Post-partum, Family planning, Kenya, Zambia

## Abstract

**Background:**

It is often assumed, with little supportive, empirical evidence, that women who use maternal health care are more likely than those who do not to use modern contraceptives. This study aims to add to the existing literature on associations between the use of antenatal (ANC) and post-natal care (PNC) and post-partum modern contraceptives.

**Methods:**

Data come from the most recent Demographic and Health Surveys (DHS) in Kenya (2008–09) and Zambia (2007). Study samples include women who had a live birth within five years before the survey (3,667 in Kenya and 3,587 in Zambia). Multivariate proportional hazard models were used to examine the associations between the intensity of ANC and PNC service use and a woman’s adoption of modern contraceptives after a recent live birth.

**Results:**

Tests of exogeneity confirmed that the intensity of ANC and PNC service use and post-partum modern contraceptive practice were not influenced by common unobserved factors. Cox proportional hazard models showed significant associations between the service intensity of ANC and PNC and post-partum modern contraceptive use in both countries. This relationship is largely due to ANC services; no significant associations were observed between PNC service intensity and post-partum FP practice.

**Conclusions:**

While the lack of associations between PNC and post-partum FP use may be due to the limited measure of PNC service intensity, the study highlights a window of opportunity to promote the use of modern contraceptives after childbirth through ANC service delivery. Depending on the availability of data, further research should take into account community- and facility-level factors that may influence modern contraceptive use in examining associations between ANC and PNC use and post-partum FP practice.

## Background

Despite the vast body of literature on modern contraceptive use, it is often assumed, with little supportive, empirical evidence, that women who use maternal health care are more likely than those who do not to use modern contraceptives. The association between maternal health care and contraceptive use is plausible for several reasons. First, family planning (FP) services are often provided within the context of maternal and child health care; therefore women who access these services may likely be exposed to FP counseling and promotion efforts. This mechanism may be particular relevant for women with a high risk pregnancy as health care providers may emphasize post-partum contraception to avoid subsequent pregnancy and health risks [1]. Second, as a woman obtains maternal and child health care, she may develop trust with the health care system. This trust can help remove social barriers to accessing FP services and provide motivations for her to use multiple services from the system. Such an effect is independent of whether FP services are included in maternal health care packages. In addition, a woman’s early contact with the health care system may also reduce cognitive, psychosocial, and indirect financial barriers - in the forms of time and opportunity costs - to subsequent FP service use. Finally, the use of maternal and child health care likely contributes to improved infant and child survival, motivating mothers to seek and use FP methods.

A surprisingly limited number of research studies have examined linkages between the use of maternal health care, namely antenatal care (ANC), delivery, and postnatal care (PNC), with contraceptive use after a child birth [2,3]. Results of these studies are mixed in terms of whether maternal health care would lead to FP use after childbirth, or whether women’s use of maternal health care and FP services is determined by some common factors. Zerai and Tsui [4] reported a strong influence of ANC use on subsequent use of modern contraception in Bolivia, Egypt, and Thailand. The evidence in Bolivia and Egypt also indicated that ANC was an immediate pathway for socio-demographic and other individual-level characteristics to influence contraceptive use, whereas in Thailand, ANC use was not necessarily required to facilitate contraceptive use [4].

More recently, Hotchkiss et al. [5] examined this topic in five countries: Bolivia, Guatemala, Indonesia, Morocco, and Tanzania. Unlike in Zerai and Tsui [4], where a dichotomous indicator of ANC usage was used, a continuous index of the intensity of maternal and child health (MCH) service use was constructed based on a series of questions related to ANC, delivery care, and child vaccination in Hotchkiss et al. [5]. In Morocco, Guatemala, and Indonesia, the evidence suggested that the use of MCH services might have served as a “gateway” to FP use [5]. In the other two countries, however, the authors found that positive associations between MCH service use and FP practice were best explained by observed and unobserved factors that might have predisposed women to both practices [5]. Evidence in these two countries is consistent with earlier research findings [2,3].

This current study aims to add to the body of evidence on the associations between maternal health care (ANC and PNC services in particular) and FP practice. It seeks to answer the question of whether the use of modern FP methods after a childbirth is associated with the use of antenatal (ANC) and postnatal care (PNC) relating to that childbirth. The relationships will be examined for the combined intensity of ANC and PNC services, as well as for ANC services and PNC services separately.

## Methods

### Data

This study focuses on Kenya and Zambia, selected for the following reasons: 1) each country has a Demographic and Health Survey (DHS) conducted in 2007 or later; 2) the DHS included a birth and contraceptive calendar; and 3) there was substantial contraceptive use (prevalence of 20% or more). The criteria are used to ensure that the study samples will include sufficiently large numbers of contraceptive users after the most recent childbirth to allow meaningful analyses. Kenya and Zambia also have similar program contexts. Public health services in Kenya and Zambia rely heavily on external funding and service delivery is often vertically segregated [6]. Consequently, maternal health care and FP programs are overseen by different bodies within and outside of the Ministry of Health, with separate sets of service standards and guidelines [7,8].

At the time of this study, the most recent DHS was conducted in 2008–09 in Kenya and 2007 in Zambia. Data for this secondary analysis was downloaded from the MEASURE DHS website with approval from MEASURE DHS. Both are based on nationally representative samples of households, men, and women of reproductive age and collected up-to-date information on a number of demographic and health indicators, including: fertility, mortality, FP, maternal and child health, and HIV/AIDS. Details of the sampling procedure can be found in each country’s DHS final report [9,10]. Data used in this analysis come from information collected with the Woman’s Questionnaire. Only women who had a live birth within five years before the survey were included in this study, resulting in study samples of 3,667 women in Kenya and 3,587 women in Zambia.

### Outcome

The outcome of interest is the use of modern contraceptive methods after the last childbirth. Information comes from the birth and contraceptive use calendar, included in the DHS Women’s Questionnaire, which records month-by-month all events related to pregnancy, pregnancy outcomes, childbirth, breastfeeding, and contraceptive use for 60 months before the survey. The outcome is measured by duration (in months) from the time of the last childbirth to the time that a woman started using a modern method of contraception. At the time of the survey, if a woman had not adopted any modern contraceptive method, she is considered a censor, for whom we do not have information on whether and when she would adopt a modern method of contraception.

### Independent variables

The main independent variable of interest is the use of ANC and PNC services relating to the last childbirth within five years before the survey. The following questions and categories are used to construct this variable:

1. Whether a woman used ANC services and if so, when she had the first visit (non-use of ANC services, first trimester, second trimester, or last trimester);

2. The number of ANC visits (less than 4 versus 4 or more visits);

3. Whether a woman received tetanus vaccine during her pregnancy (yes versus no);

4. Whether ANC services were provided by a trained provider, including doctors, nurses, and midwives (yes versus no);

5. Whether a woman received the following ANC procedures, including measuring weight, height, blood pressure, taking urine sample and blood sample, breastfeeding counseling, and being told about signs of complications (yes versus no, 7 items);

6. Whether a woman received PNC checkup before being discharged, soon after discharge, and within two months after the live birth and if so, whether it was provided by a trained provider, including: doctors, nurses, and midwives (yes versus no).

It should be noted here that a potential limitation is the limited information on PNC services compared to ANC services available from the DHS. Details, including the content and quality of PNC services, as well as whether it is a woman’s choice to receive PNC services, are not asked in the DHS. Dummy variables were created to indicate binary responses to each question or category.

Principal component analysis was used to construct scores that measure the intensity of both ANC and PNC services, as well as the intensity of ANC and PNC services separately. The internal reliability coefficient of these constructs ranges from .77 to .82 in Kenya, and from .50 to .71 in Zambia, indicating a reasonable to high level of correlation between the items used. These continuous service intensity variables were used as the main predictors in the analyses. It is worthy to note that among the indicators used to construct the ANC intensity score, several factors are considered within the control of women (including the timing and number of ANC visits), while the others are unlikely for women to have control over (including specific ANC procedures that women receive). The latter is likely dependent on the capacity and quality of services provided within the health system. Therefore, we conducted the analysis with separate ANC service intensity scores for those within and outside of the control of women. If the former factors are found to be associated with the FP use outcome, intervention efforts should target individual women; if the latter factors are related to post-partum FP use, efforts to promote FP use may be successful if targeting ANC service delivery.

Other controlling variables include women’s basic socio-demographic characteristics and several variables that may influence contraceptive use. Factors that are hypothesized to directly affect contraceptive in this study include: knowledge of contraceptive methods (measured as the number of modern contraceptive methods known), whether a woman was visited and talked about FP by a field worker in the last 12 months before the survey, whether a woman visited and talked about FP at a health facility in the last 12 months, desire for more children, use of any modern contraceptive method prior to the index^a ^childbirth, and whether a woman recalled a FP message in the mass media (TV, radio, and newspapers). Age of the women at their first childbirth may indicate potentially greater risks of pregnancy complications and was hypothesized to be related to ANC use. Durations of breastfeeding and post-partum^b^ amenorrhea were also controlled for as they may influence women’s decision to start using contraceptives.

### Methods

Cox proportional hazard model was employed to examine the time duration from the last childbirth to a woman’s adoption of a modern contraceptive, as well as factors influencing this interval. Multivariate models were used to assess associations between ANC and PNC service intensity and the outcome, controlling for potential confounders.

Because there is a possibility that ANC and PNC service utilization is endogenous to post-partum modern FP use (i.e. they are determined by the same observed and unobserved women’s characteristics), test of exogeneity was performed. We followed the procedure laid out by Bollen, Guilkey and Mroz [11], which involved estimating two equations: the first equation is an ordinary least square estimation of ANC and PNC service intensity score; the second equation is a proportional hazard model, in which the error term obtained from the first equation is included with the actual service intensity score. If the hazard ratio associated with the error term is not significantly different from zero, one would accept the null hypothesis that the ANC/PNC service intensity is exogenous in the contraceptive use equation. On the other hand, if the hazard ratio of the error term is statistically significant from zero, there is evidence of endogeneity and a two-step equation system should be used.

This two-equation procedure also requires ANC/PNC service intensity and post-partum modern FP use to be identified by distinct variables or sets of variables, although some of the determinants may overlap [11]. These are instrumental variables that are theoretically related to ANC/PNC service intensity and not related to post-partum modern contraceptive use, or vice versa. In this study, the age of women at first birth was hypothesized to present pregnancy risks and to be related to ANC/PNC services only. Several factors, including the desire for more children, knowledge of modern contraceptives, visits by a FP field worker as well as to a health facility, as well as exposure to FP messages in the media (TV, radio, and newspapers) were hypothesized to be directly associated with only post-partum modern FP use. Hausman specification and log-likelihood ratio tests were used to examine whether the exclusion of these variables from the respective equations was appropriate.

In both countries, the test of exogeneity showed no statistically significant associations between post-partum modern FP use and the error term of ANC/PNC service intensity. In Kenya, the p-value associated with the hazard ratio of the error term of ANC/PNC service intensity is .42; in Zambia, it is .06; both indicating that the error term was not significantly associated with post-partum modern FP use. Specification tests also confirmed that the exclusion of the instrumental variables did not make a difference to the respective equations (results not shown). Therefore, the intensity of ANC/PNC service use can be employed as a predictor in the proportional hazard model for post-partum modern FP use.

## Results and discussion

The following sections present findings from each country. For the purpose of this study, the discussion focuses on factors that influence post-partum modern contraceptive use, although regression results for ANC/PNC service intensity are also presented in the tables.

### Kenya

#### Country background

Until the first Demographic and Health Survey (DHS) in 1989, Kenya had one of the highest birth rates in the world and experienced high annual population growth rates. FP services were then integrated with maternal and child health (MCH) services but the development of the program was relatively slow [12]. The FP program became multi-sectoral in the 1980s, with increasing roles of the private sector [13]. Nevertheless, the government continues to be the major provider of contraceptives, particularly as most private and NGOs switched to HIV/AIDS activities because of the shift of funding priorities by major donors: 53 percent of all modern method users in 2003 obtained contraceptives from a public sector source [14,15].

The use of ANC is widespread: more than 90% of pregnant women received ANC from a trained medical provider in 2008–09, a slight increase from 2003 [9,14]. The average number of ANC visits per pregnancy is about 4; yet, women often obtain ANC later in the pregnancy: the median pregnancy duration at the first ANC visit is about 5 months [9,16]. We are not aware of any analysis based on the Kenya DHS 2008–09, but an analysis of the earlier DHS (2003) found a positive association between the number of ANC visits and post-partum FP use [17]. However, discussions of FP during ANC visits are rare [8,18]. Results from a recent pilot introduction of an integrated service package showed that the integration was acceptable to both clients and providers [8]; yet currently there are no standards or guidelines for the integration of these services.

PNC use is much less common compared with the use of ANC services, in part due to a lack of attention and support for post-partum care among both providers and the public [19]. In 2008–09, just under half of women received any PNC after a recent childbirth with no changes compared to 2003 [9,14]. The under-utilization of PNC presents missed opportunities for FP discussion as many Kenyan women reported that PNC visit would be a good time for such a discussion and FP method provision [20]. Indeed, an intervention that introduced a package of PNC and FP services in Kenya has shown to result in substantial improvement in post-partum FP uptake, as well as earlier initiation of FP use after a childbirth [21]. Few other research studies in Kenya have also suggested that FP services integrated with MCH services might result in increased contraceptive use and fertility decline, even in rural areas [22,23].

#### Findings

**Figure 1:**
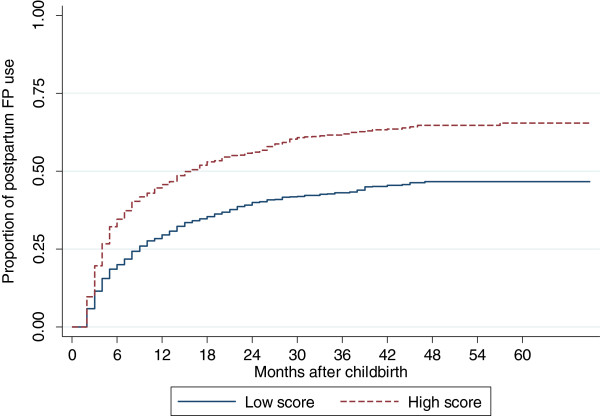
Rates of post-partum FP adoption by ANC and PNC service intensity score among women who had a childbirth within the last five years, Kenya, 2008–2009.

Forty-six percent (1,689) of the Kenyan sample had adopted a modern method of contraception at some point between the last childbirth and the time of the survey. The median time between childbirth and modern contraceptive adoption was 5 months among users. To aid the visual description of post-partum contraceptive use, Figure 1 displays the rate of FP adoption stratified by high and low scores of the ANC and PNC service intensity (cut-off point is the median score).

This figure shows that women who had a high score of ANC/PNC service intensity consistently adopted modern contraceptives earlier and at a higher rate, compared to women with a low ANC/PNC service intensity score. In both groups, contraceptive adoption increased quickly in the first six months after childbirth. In the high service intensity score group, as many as one in four women started using a modern contraceptive within the first 6 months post-partum. The rates of contraceptive adoption slowed down markedly in both groups between 6 and 18 months after childbirth, and plateaued after this point.

Column 1 of Table 1 presents the distribution of the Kenyan study sample. The vast majority (93%) of women in the sample received ANC services; however, Kenyan women initiated ANC fairly late during the pregnancy: 61.3% of them did not initiate ANC until the second trimester; another 15.9% started getting ANC only in the last trimester. More than half (53%) of the Kenyan sample did not meet the WHO’s standard of 4 ANC visits during a pregnancy. PNC service use was much less frequent compared with ANC services: only 48.8% of women in the sample received any PNC after the index childbirth.

**Table 1:** Factors influencing ANC/PNC use and post-partum modern FP use among women who had a childbirth within the last five years, Kenya, 2008–09

**Characteristic**	**Distribution % or mean (95% C.I.)**	**ANC/PNC service intensity**	**Post-partum modern FP use**
		**Coef. (95% C.I.)**	**Hazard ratio (95% C.I.)**^**1**^
	**(1)**	**(2)**	**(3)**
Median duration from childbirth to modern contraceptive adoption (months)	5		
Timing of first ANC visits			
None	7.67		
1st trimester	15.11		
2^nd ^trimester	61.36		
3^rd ^trimester	15.86		
Had more than 4 ANC visits			
No	52.96		
Yes	47.04		
Use of PNC services			
No	51.19		
Yes	48.81		
ANC and PNC service intensity score	−.02 (−1.22; 1.13)	−	1.09 (1.01; 1.18)*
Urban	20.98	.17 (.06; .27)***	.96 (.82; 1.14)
Region			
Nairobi	8.64	−	1.00
Central	9.79	−.08 (−.23; .08)	1.37 (1.10; 1.71)**
Coast	14.70	.20 (.05; .36)*	1.03 (.83; 1.29)
Eastern	13.01	−.13 (−.28; .02)	1.12 (.91; 1.46)
Nyanza	17.32	−.22 (−.36; -.07)**	.81 (.65; 1.01)
Rift Valley	16.85	−.17 (−.31; -.03)*	.86 (.69; 1.07)
Western	12.30	−.19 (−.34; -.04)*	1.16 (.93; 1.45)
Northeastern	7.39	−.41 (−.65; -.16)	.27 (.14; .54)***
Age group			
15 – 19	6.35	−	1.00
20 – 24	26.78	.06 (−.06; .18)	1.15 (.89; 1.47)
25 – 29	26.83	.10 (−.03; .23)	1.05 (.81; 1.36)
30 – 34	20.28	.04 (−.10; .17)	.88 (.67; 1.15)
35 – 39	11.54	−.09 (−.23; .06)	.76 (.59; 1.01)
40 – 49	8.21	−.19 (−.35; -.04)*	.59 (.43; .82)**
Highest education level			
No education	10.64	−	1.00
Primary school	62.08	.45 (.34; .56)***	1.95 (1.49; 2.56)***
Secondary school or higher	27.33	.57 (.44; .70)***	2.12 (1.58; 2.84)***
Wealth quintile			
Poorest	20.51	−	1.00
Poor	19.06	.10 (.01; .19)*	1.44 (1.19; 1.75)***
Middle	18.69	.13 (.04; .22)**	1.41 (1.17; 1.71)***
Rich	19.73	.12 (.02; .21)*	1.52 (1.25; 1.85)***
Richest	22.02	.11 (−.01; .23)	1.52 (1.20; 1.92)**
Religion			
Catholic	20.56	−	1.00
Protestant/Other Christians	68.54	−.01 (−.08; .06)	1.00 (.88; 1.13)
Muslim/Others	10.90	−.23 (−.35; .-10)***	.83 (.67; 1.03)
Marital status			
Not married	18.79	−	1.00
Married, monogamous	68.18	.19 (.11; .26)***	2.12 (1.84; 2.45)***
Married, polygamous	13.04	.15 (.04; .26)**	1.55 (1.26; 1.92)**
Work in the last 12 months			
No	39.14	−	1.00
Yes	60.86	−.03 (−.09; .03)	1.18 (1.06; 1.31)**
Age at first birth	19.18 (14; 37)	.02 (.01; .02)**	−
Use of modern contraceptives prior to last childbirth			
No	53.64	−	1.00
Yes	46.36	.15 (.09; .21)***	1.53 (1.37; 1.70)***
Desire for more children			
No	50.30	−	1.00
Yes	49.70		.90 (.80; .99)*
Knowledge of modern contraceptives (range: 0–10)	6.72 (0; 10)	−	1.06 (1.03; 1.08)***
Visited and talked about FP by a field worker in the last 12 months			
No	91.92	−	1.00
Yes	8.08		.88 (.74; 1.06)
Visited and talked about FP at a health facility in the last 12 months			
No	70.03	−	1.00
Yes	20.97		1.19 (1.06; 1.35)**
Heard FP messages on the radio in the last few months			
No	28.45	−	1.00
Yes	71.55		1.15 (1.01; 1.33)*
Saw FP messages on TV in the last few months			
No	60.96	−	1.00
Yes	35.04		1.02 (.89; 1.18)
Read FP messages in newspapers in the last few months			
No	69.44	−	1.00
Yes	30.56		.91 (.79; 1.05)
N	3,667		

Among women’s characteristics, it is noteworthy that a small, but significant proportion (13%) were living in a polygamous marriage. A substantial proportion (18.8%) of women who recently gave birth were not currently married. Women were 19 years old on average at their first birth. About half of the study women wanted to have more children after the index childbirth. Less than half (46.4%) had used a modern method of contraception prior to the index birth. Women in the sample knew 6.72 methods of contraception on average. Most (91.92%) of them were not visited by a FP field worker within the last 12 months; the majority (70%) also never visited and talked about FP at a health facility during the same time period.

##### ANC/PNC service intensity and post-partum modern contraceptive use

Results shown in column 2 of Table 1 indicate several factors that were significantly related to ANC and PNC service use, including: urban residence, education, household wealth, marital status, prior use of modern contraceptives, and age at first birth. It is important to note that women who ever used a modern method of contraception before the conception of the index child used ANC/PNC services more intensively than did others (p<.001).

Column 3 presents results of the proportional hazard model, predicting post-partum modern contraceptive use, controlling for factors that may influence contraceptive use behaviors. ANC/PNC service intensity was significantly related to the contraceptive use outcome. After controlling for other factors that may influence FP use, an increased ANC/PNC intensity score was significantly associated with a moderate but significant increase in the likelihood of modern FP use after a woman’s last birth (hazard ratio=1.09; p<.05). We also found that the use of any modern contraceptives prior to the index childbirth was significantly associated with ANC service use, as well as with post-partum contraceptive use. It indicates that the possibility that the use of ANC services was a result of earlier contacts with the health system for FP services cannot be ruled out. A longitudinal, prospective study design may be necessary to assess the temporality of the relationship.

We also examined the relative importance of the intensity of ANC and PNC services to post-partum FP use separately following the same procedures. Tests of exogeneity confirmed that the two intensity scores for ANC and PNC services can be used as predictors of post-partum modern FP use. Table 2 shows partial results of the multivariate proportional hazard model where two intensity scores were used for ANC and PNC services instead of the composite intensity score. These results indicate a significant, positive association between ANC service intensity and post-partum modern contraceptive use (p<.05). PNC service intensity was not found significantly related to post-partum modern contraceptive use, likely due to the limited measure of PNC service available from the data. The associations between controlling factors and contraceptive use behavior remained the same as in column 3 of Table 1 (discussed below).

**Table 2:** Influence of ANC and PNC services on post-partum modern FP use, Kenya, 2008–09

**Characteristic**	**Distribution**	**Post-partum modern FP use**
	**% or mean (s.e.)**	**Hazard ratio (s.e.)**^**1**^
	**(1)**	**(2)**
ANC service intensity score	.06 (−2.72; .88)	1.09 (1.01; 1.17)*
PNC service intensity score	.70 (0; 2)	1.01 (.92; 1.12)
N	3,667	

^1^Model controls for all women’s characteristics included in Table 1.

† p<.010; * p<.05; ** p<.01; *** p<.001.

The supplementary analysis (results not shown) showed that only the ANC service intensity constructed based on factors that are outside of women’s control was associated with post-partum modern FP use. The PNC service intensity score was still not related to the FP outcome. The finding suggests that improving the quality of ANC services may be an important way to promote modern FP use after a childbirth.

##### Other factors associated with post-partum modern contraceptive use

Among basic socio-demographic factors, women’s education, household wealth, marital status, and women’s employment in the last 12 months consistently had significant, positive associations with post-partum modern contraceptive use. Other important predictors of post-partum modern FP use among this Kenyan sample include: desire for more children, previous use of modern contraceptives prior to the index birth, knowledge of contraceptives, visits to a health facility within the last 12 months, as well as listening to FP messages on the radio.

Previous use of modern contraception was a strong, positive predictor of post-partum modern FP use: women who used a modern contraceptive prior to the index birth were 1.53 times as likely as those who did not to adopt a modern method of contraception post-partum (p<.001). Modern contraceptive use after the last childbirth was also positively associated with knowledge of contraceptive methods and visits to a health facility. An increase of one method of contraception known to a woman was related to a small but significant increase of post-partum contraceptive use (hazard ratio=1.06; p<.001). The likelihood of using modern contraceptives after the last childbirth was also significantly increased among women who visited and talked about FP at a health facility within the last 12 months, compared to women who did not (hazard ratio=1.19; p<.01). Radio seemed to be the most effective medium for FP messages in this sample: having heard a FP message on the radio was related to a significantly increased likelihood of using modern contraceptives (hazard ratio=1.15; p<.05). As expected, desire for more children after the index childbirth was a negative predictor of post-partum contraceptive use. Compared to women who wanted no more children, those who wanted more children were only .90 times as likely to adopt a modern contraceptive post-partum (p<.05).

### Zambia

#### Country background

Population growth was not deemed a development issue by the Zambian government until the issue of the Fourth National Development Plan in 1989–1993, which included a national population policy aiming to reduce TFR from 7.2 to 6 and to make FP services accessible and affordable to at least 30% of those in need [24]. Currently, only one-third of married women are using modern contraceptives [10].

Similarly to Kenya, the use of ANC services in Zambia is high, even in rural areas [6,25,26]. Many women have five or more ANC visits during a pregnancy [26,27]. Despite a high level of client satisfaction, direct observations indicated poor quality of ANC services: just over half of ANC clients received any health education; in addition, ANC attendance did not influence the use of other maternal health care, such as facility-based delivery [25].

While it is recommended that mothers obtain PNC at one and six weeks after delivery, the use of PNC services remains somewhat low and varies widely. In one study in Lusaka, as many as 84% of women reported a postnatal checkup within the first six weeks after birth [26]. In rural areas, the proportion of PNC use within 6 weeks of delivery could be as low as 42% [28]. PNC often receives much less attention than ANC and delivery care [29]. Many women did not know about the existence of PNC or thought that it was only necessary if they had pregnancy complications [30]. Lagro et al. [28] also reported that women who gave birth at home felt unwelcome to attend post-partum clinics. Additionally, because services are often not well integrated, providers often miss the opportunities for PNC service delivery when women visit a health facility for other services, such as child vaccination [28].

#### Findings

Of the 3,587 Zambian who had given childbirth within five years before the survey, 1,647 (45.9%) adopted a modern method of contraception within the observation period. The median time of adoption was 8 months after the last childbirth.

**Figure 2:**
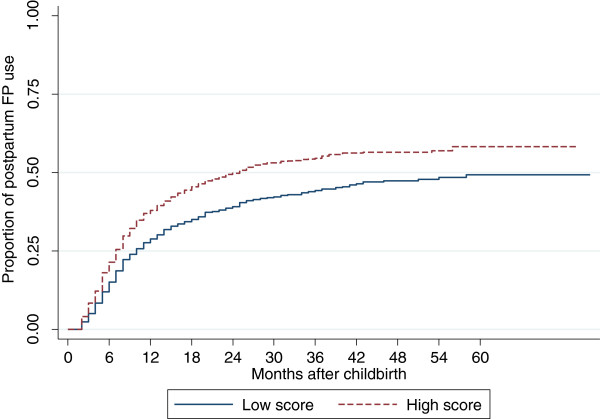
Rates of post-partum FP adoption by ANC and PNC service intensity score among women who had a childbirth within the last five years, Zambia, 2007.

Figure 2 shows the rate of modern contraceptive adoption among Zambian women who had given birth within 5 years before the survey, by the score of ANC/PNC service intensity. Women who had a low ANC/PNC service score consistently adopted modern contraceptives at a lower rate than women with a high service score did. For both groups, modern contraceptive adoption seemed gradual in the first 18 months after childbirth and reached the plateau at this point.

Column 1 of Table 3 presents the distribution of the Zambian study sample. Nearly every woman (97.5%) used ANC services for the index childbirth; yet, the majority (78%) did not initiate ANC visits until the second trimester or later. About 60% of women in the Zambian sample had at least 4 ANC visits during the index pregnancy. Only half (51.1%) of the study women used any PNC services for the index childbirth.

**Table 3:** Factors influencing ANC/PNC use and post-partum modern FP use among women who had a childbirth within the last five years, Zambia, 2007

**Characteristic**	**Distribution % or mean (95% C.I.)**	**ANC/PNC service intensity**	**Post-partum modern FP use**
		**Coef. (95% C.I.)**	**Hazard ratio (95% C.I.)**^**1**^
	**(1)**	**(2)**	**(3)**
Median duration from childbirth to modern contraceptive adoption (months)	8		
Timing of first ANC visits			
None	2.56		
1st trimester	19.57		
2^nd ^trimester	70.03		
3^rd ^trimester	7.85		
Had more than 4 ANC visits			
No	40.09		
Yes	59.91		
Use of PNC services			
No	48.84		
Yes	51.16		
ANC and PNC service intensity score	−.02 (−1.22; 1.13)	−	1.09 (1.01; 1.16)*
Urban	33.65	.25 (.15; .36)***	.92 (.78; 1.08)
Region			
Central	9.97	−	1.00
Copperbelt	15.23	.21 (.09; .33)**	1.17 (.95; 1.46)
Eastern	15.21	.35 (.23; .46)***	.79 (.63; .98)*
Luapula	7.81	.06 (−.07; .19)	.67 (.51; .87)**
Lusaka	12.74	.35 (.22; .48)	1.07 (.87; 1.33)
Northern	14.90	−.08 (−.19; .04)	1.30 (1.06; 1.61)*
Northwestern	5.65	.03 (−.12; .18)	1.23 (.99; 1.54)
Southern	11.01	.03 (−.09; .15)	1.11 (.90; 1.36)
Western	7.48	.33 (.19; 47)***	1.06 (.85; 1.32)
Age group			
15 – 19	8.70	−	1.00
20 – 24	24.24	.04 (−.08; .15)	1.03 (.83; 1.28)
25 – 29	26.56	−.02 (−.13; .10)	.93 (.74; 1.16)
30 – 34	19.15	.01 (−.12; .13)	.88 (.69; 1.13)
35 – 39	11.86	.11 (−.03; .24)	.75 (.58; .99)*
40 – 44	9.49	.02 (−.12; .16)	.54 (.40; 73)***
Highest education level			
No education	13.12	−	1.00
Primary school	60.19	.29 (.20; .37)***	1.33 (1.10; 1.60)**
Secondary school or higher	26.69	.41 (.31; .52)***	1.55 (1.25; 1.91)***
Wealth quintile			
Poorest	21.73	−	1.00
Poor	20.88	.05 (−.03; .14)	.97 (.81; 1.16)
Middle	19.64	.16 (.07; .25)***	1.06 (.89; 1.26)
Rich	21.13	.31 (.18; .43)***	1.28 (1.03; 1.58)*
Richest	16.62	.31 (.15; .46)***	1.52 (1.17; 1.96)**
Religion			
Catholic	19.32	−	1.00
Protestant/Other Christians	78.76	.01 (−.06; .08)	1.02 (.88; 1.13)
Muslim/Others	1.92	−.64 (−.85; -.42)***	.84 (.55; 1.28)
Marital status			
Not married	20.35	−	1.00
Married, monogamous	67.79	−.02 (−.09; .06)	2.66 (2.29; 3.08)***
Married, polygamous	11.86	−.10 (−.21; .01)	2.32 (1.88; 2.86)***
Work in the last 12 months			
No	43.13	−	1.00
Yes	56.87	.01 (−.05; .07)	1.15 (1.04; 1.28)**
Age at first birth	18.60 (15; 36)	.01 (−.01; .01)	−
Use of modern contraceptives prior to last childbirth			
No	47.47	−	1.00
Yes	52.53	.02 (−.04; .09)	1.26 (1.13; 1.41)**
Desire for more children		−	
No	33.64		1.00
Yes	66.36		.96 (.85; 1.09)
Knowledge of modern contraceptives (range: 1–11)	6.77 (3; 11)	−	1.04 (1.01; 1.07)**
Visited and talked about FP by a field worker in the last 12 months		−	
No	92.19		1.00
Yes	7.81		1.18 (1.00; 1.39)
Visited and talked about FP at a health facility in the last 12 months			
No	67.56		1.00
Yes	32.44		1.29 (1.16; 1.44)***
Heard FP messages on the radio in the last few months		−	
No	61.88		1.00
Yes	38.12		1.11 (.99; 1.24)
Saw FP messages on TV in the last few months		−	
No	83.54		1.00
Yes	16.46		.92 (.78; 1.09)
Read FP messages in newspapers in the last few months		−	
No	91.81		1.00
Yes	8.19		1.03 (.85; 1.24)
N	3,587		

^1^Model controls for durations of breastfeeding and amenorrhea.

† p<.010; * p<.05; ** p<.01; *** p<.001.

It should be noted that while the majority of sampled women were in a monogamous marriage, one in five were not married at the time of the survey; a moderate proportion (11.9%) were in a polygamous marital relationship. The mean age at first birth was 18.6 for women in this sample. Two-thirds of them wanted to have more children after the index birth. Just over half of the women (52.5%) had used a modern method of contraception prior to the index birth. The mean number of modern contraceptive methods known to women in the sample was 6.8. The vast majority of them (92.2%) were not visited by a FP field worker in the 12 months before the survey; about two-thirds never visited and talked about FP at a health facility within 12 months before the survey.

##### ANC/PNC service intensity and post-partum modern contraceptive use

Column 2 of Table 3 shows several women’s characteristics that are positively related to the use of ANC and PNC services, including: urban residence, women’s education, and household wealth. Unlike Kenya, no association was observed between women’s prior use of modern contraceptives and ANC/PNC service use intensity.

Column 3 presents results of the multivariate proportional hazard model. ANC/PNC service intensity score is shown to have a significant, positive association with post-partum modern FP use. The result indicates that after the confounders were controlled for, an increase of one point in the service intensity score was associated with a 9 percentage point increase in the likelihood of post-partum modern FP use (p<.05). When ANC and PNC services were separated in the multivariate model, as shown in Table 4, we found a similar significant, positive association between the ANC service intensity and post-partum modern contraceptive use (hazard ratio=1.08; p<.05). Like in Kenya, the PNC service intensity score was not shown in Zambia to have a significant association with post-partum modern FP practice, possibly because of limitations in its measurement.

**Table 4:** Influence of ANC and PNC services on post-partum modern FP use, Zambia, 2007

**Characteristic**	**Distribution**	**Post-partum modern FP use**
	**% or mean (s.e.)**	**Hazard ratio (s.e.)**^**1**^
	**(1)**	**(2)**
ANC service intensity score	.02 (−1.66; 1.26)	1.08 (1.02; 1.16)*
PNC service intensity score	.61 (.0; 2)	.99 (.91; 1.09)
N	3,587	

^1^Model controls for all women’s characteristics included in Table 1.

† p<.010; * p<.05; ** p<.01; *** p<.001.

Similarly to Kenya, the supplementary analysis in Zambia also shows associations between post-partum FP use with only the ANC service intensity score constructed based on factors that are outside of the control of women. PNC service intensity remained unrelated to FP use in the analysis. The findings in both countries underline the importance of improving the quality of ANC service delivery in order to increase the use of contraception after a childbirth. Furthermore, as the vast majority of women in our study obtained ANC services from the public sector (82% in Kenya and 92% in Zambia), the findings underline the needs for the integration of ANC and FP services within the public sector in order to take advantage of the pregnancy period as a window of opportunity to promote FP use.

##### Other factors associated with post-partum modern contraceptive use

The section below briefly discusses the associations between other women’s characteristics and post-partum modern FP use as shown in column 3 of Table 3. These associations did not change whether the composite measure or two separate measures of ANC and PNC service intensity were used in the regressions.

Post-partum modern contraceptive use was positively associated with women’s education, marital status, and women’s work in the last 12 months. In addition, previous use of modern contraceptives, contraceptive knowledge, as well as visits by a FP field worker and to a health clinic in the 12 months before the survey had positive associations with modern FP use after the last childbirth. Women who had used a modern method of contraception before the index childbirth were more likely than those who had not to adopt a modern contraceptive post-partum (hazard ratio=1.26, p<.01). An increase of a modern method of contraception known to a woman was also associated with a small but significant increase in the likelihood of modern contraceptive use (p<.01). Similarly to Kenya, visits to a health center were associated with an increased likelihood of modern contraceptive use after a woman’s last childbirth. Women who visited and talked about FP at a health clinic were 1.29 times as likely as those who did not to use a modern method of contraception after the last childbirth (p<.001).

## Conclusions

This study examines the associations between the use of maternal health care (specifically ANC and PNC services) and post-partum modern FP practice in Kenya and Zambia. Results indicate a positive association between the intensity of ANC and PNC services and post-partum use of modern contraception in both countries. The more intensively women use ANC and PNC services, the more likely they go on to adopt a modern method of contraception after the index childbirth. The evidence also suggests that the use of maternal health services can be a mediator for individual’s socio-demographic characteristics to influence post-partum modern contraceptive use. Tests of exogeneity indicate no evidence that maternal health care use and post-partum FP practice were influenced by common unobserved factors. In addition, we found that when maternal health care was disaggregated into ANC and PNC services, only the use of ANC services was found to be significantly related to post-partum modern contraceptive use.

An important program implication of the findings is that the promotion of ANC services should be considered as a mechanism to promote post-partum FP use. Since the use of contraception after a childbirth is related to ANC service factors that are relating to health providers and the health system, a health system approach to improving ANC services should be a priority in low-resource settings. Policy makers in countries like Kenya and Zambia, where most women already receive at least one ANC visit, who want to promote post-partum FP use should emphasize the comprehensiveness and quality of ANC services. For example, women should be made sure to receive tetanus vaccine and a range of standard ANC procedures. Such services may contribute to women’s trust of the health system and their overall satisfaction with health care services, which make them more likely to return for other services. As most women who attend ANC clinics do not regularly receive any health education [25], the provision of FP counseling during ANC visits may also serve to improve the quality of these visits and client satisfaction. In fact, previous research suggests that a pilot introduction of a focused ANC package that included FP counseling in two districts in Kenya was welcomed by providers as well as clients and significantly increased the overall quality of care [8].

The null finding related to PNC service intensity warrants some discussion. Theoretically, the use of PNC should be related to post-partum FP practice for the same reasons that ANC is related to this contraceptive behavior outcome. Previous research in the sub-Saharan African region has shown that PNC is among the weakest aspects of reproductive health programs [20,21,31]. Health facilities do not routinely record PNC visits; the vast majority of women who deliver their babies at home do not receive PNC [19]. In addition, although FP counseling is theoretically part of the routine PNC package, in practice it is often overlooked when priority is given to a child’s health during postnatal checkups [28]. Even when PNC services are used by women, they still seem to be a missed opportunity for FP promotion; for example, 68% of post-partum women in Kenya had unmet need for FP in during the first year [32]. Moreover, access to PNC services remains limited, at least in the two countries under this study [28].

However, because we cannot test these hypotheses with the available DHS data, it is likely that the null finding related to PNC service in this study is due to limitations in the measure of PNC services available in the DHS. There are no questions about the content or quality of PNC services. Our measure of PNC service intensity is only based on three binary indicators of whether a woman received any check-up by a trained provider before, after discharge and two months after the childbirth; the first one is likely outside of women’s control. Therefore, it is possible that the quality of PNC services, for which we do not have a measure, may be related to post-partum FP use. Had we had more information on the content and quality of PNC services, the results might have been different.

In addition to ANC and PNC service use intensity, several other individual characteristics that are important for FP practice are highlighted in this study. As mentioned earlier, prior use of modern contraception has a direct association with post-partum FP use, as well as an indirect one through ANC service intensity in Kenya. It is important to note that in both countries, women who visited and talked about FP at a health facility within the last 12 months were significantly more likely than others to adopt a modern contraceptive after a childbirth. The finding further underlines the role of the health care system in promoting FP practice. Socio-economic characteristics, including education, household wealth, and women’s employment, were also consistently associated with an increased likelihood of post-partum modern contraceptive use, suggesting that efforts to increase FP use should continue to address women of lower education level, from poorer households, and those who do not work outside of the home.

One limitation of this study is that the results are not necessarily generalizable to all women of childbearing age in Kenya and Zambia. The group of women included in this study, married and cohabiting women who gave birth within five years before the survey, are significantly different from women who were not included in terms of a number of socio-demographic factors. Study women were older, less educated, poorer, and more likely to live in rural areas than those who were not in the study (results not shown).

Another potential limitation of the study is the possible endogeneity between the FP use outcome and the variables relating to exposure to FP messages on the media and visits by a FP field worker. Some women may have been motivated to adopt a modern method of contraception because of their exposure to FP messages in the media or because they were visited by a FP field worker. On the other hand, it is plausible that women who are already using contraceptives may be more likely than others to pay attention to FP messages in the media and recall them better. Contraceptive users may also be more likely than non-users to be visited by a FP fieldworker for follow up or resupply. Testing for these potential endogenous associations is beyond the scope of this study. Nevertheless, the study results did not change when these variables were excluded from the model. Therefore, any bias potentially introduced by this type of endogeneity would not significantly change our main findings.

Finally, only individual-level factors were examined in this study. It is possible that post-partum modern FP practice is influenced by community-level factors that were not measured. For example, the availability of and access to modern contraceptives in the community may influence a woman’s use of contraception. Community norms about contraceptive use may also positively influence an individual’s contraceptive behaviors. Similarly, facility-level data on the degree of integration of FP and reproductive health services would have been useful for this type of analyses. Many of these factors, however, are not readily measurable with existing DHS data.

Despite the limitations, this study adds to the currently limited body of evidence of the associations between maternal health care (and ANC service use in particular) and post-partum modern FP use, using recent nationally representative survey data in Kenya and Zambia. ANC services could provide an important opportunity to promote the use of modern contraceptives after childbirth. The findings underline the importance of working with the health system to improve ANC service delivery in order to promote post-partum modern contraceptive use, at least in the context of these two countries.

## Endnotes

^a^An index childbirth in this study is the last childbirth within five years before the survey. ^b^ The term “post-partum” is used loosely in this study to indicate the time after childbirth.

## Competing interests

The authors declare that they have no competing interests.

## Authors’ contributions

The study was conceived by MD and DH. MD conducted the analysis and drafted the manuscript. DH helped with the analysis and the manuscript. All authors have read and approved the final manuscript.

## Pre-publication history

The pre-publication history for this paper can be accessed here:

http://www.biomedcentral.com/1472-6963/13/6/prepub

## References

[B1] Cicely W Baumslag N Jelliffe D Mother and Child Health: Delivering Services 1994 New York: Oxford University Press

[B2] Ahmed S Mosley WH Simultaneity in the use of maternal-child health care and contraceptives: evidence from developing countries Demography 2002 39 1 75 93 10.1353/dem.2002.0001 11852841

[B3] Hotchkiss DR Magnani RJ Rous JJ Azelmat M Mroz TA Heikel J The effects of maternal-child health service utilization on subsequent contraceptive use in Morocco J Biosoc Sci 1999 31 2 145 165 10.1017/S0021932099001455 10333649

[B4] Zerai A Tsui AO The relationship between prenatal care and subsequent modern contraceptive use in Bolivia, Egypt and Thailand Afr J Reprod Health 2001 5 2 68 82 10.2307/3583432 12471915

[B5] Hotchkiss DR Rous JJ Seiber EE Berruti AA Is maternal and child health service use a causal gateway to subsequent contraceptive use?: A multi-country study Popul Res Policy Rev 2005 24 6 543 571 10.1007/s11113-005-4852-0

[B6] Mayhew SH Lush L Cleland J Walt G Implementing the integration of component services for reproductive health Stud Fam Plann 2000 31 2 151 162 10.1111/j.1728-4465.2000.00151.x 10907280

[B7] Lush L Service integration: an overview of policy developments Int Fam Plan Perspect 2002 28 2 71 77 10.2307/3088238

[B8] Birungi H Onyango-Ouma W Acceptability and Sustainability of the WHO Focused Antenatal Care Package in Kenya 2006 Washington, D.C.: Population Council, Frontiers in Reproductive Health

[B9] Kenya National Bureau of Statistics (KNBS), ICF Macro Kenya Demographic and Health Survey 2008–09 2010 Maryland: KNBS and ICF Macro, Calverton

[B10] Central Statistical Office (CSO), Ministry of Health (MOH), Tropical Diseases Research Centre (TDRC), University of Zambia, Macro International Inc Zambia Demographic and Health Survey 2007 2009 Calverton, Maryland: CSO and Macro International Inc

[B11] Bollen KA Guilkey DK Mroz TA Binary outcomes and endogenous explanatory variables: tests and solutions with an application to the demand for contraceptive use in Tunisia Demography 1995 32 1 111 131 10.2307/2061900 7774727

[B12] Miller RA Ndhlovu L Gachara MM Fisher AA The situation analysis study of the family planning program in Kenya Stud Fam Plann 1991 22 3 131 143 10.2307/1966641 1949097

[B13] Blacker J Opiyo C Jasseh M Sloggett A Ssekamatte-Ssebuliba J Fertility in Kenya and Uganda: a comparative study of trends and determinants Popul Stud 2005 59 3 355 373 10.1080/00324720500281672 16249155

[B14] Central Bureau of Statistics (CBS) [Kenya], Ministry of Health (MOH) [Kenya], ORC Macro Kenya Demographic and Health Survey 2003 2004 Calverton, Maryland: CBS, MOH, and ORC Macro

[B15] Family Health International (FHI) Country Assessment: Kenya. Family Planning Needs in the Context of the HIV/AIDS Epidemic 2004 Chapel Hill, N.C.: Family Health International

[B16] Magadi MA Madise NJ Rodrigues RN Frequency and timing of antenatal care in Kenya: explaining the variations between women of different communities Soc Sci Med 2000 51 4 551 561 10.1016/S0277-9536(99)00495-5 10868670

[B17] USAID, ACCESS Family Planning Needs during the Extended Postpartum Period in Kenya 2007 Washington, DC: USAID

[B18] Ministry of Health (MOH) [Kenya], National Council for Population and Development, ORC Macro Kenya Service Provision Assessment Survey 1999 2000 Calverton, Maryland: Ministry of Health, National Council for Population and Development, and ORC Macro

[B19] The Safe Motherhood Demonstration Project Repositioning Post Partum Care in Kenya Safe Motherhood 2005 Kenya: Ministry of Health, University of Nairobi, and Population Council

[B20] Warren C Daly P Toure L Mongi P Postnatal care Opportunities for Africa's Newborns 2006 Cape Town, South Africa: Partnership for Maternal, Newborn and Child Health 79 90

[B21] Mwangi A Warren C Koskei N Blanchard H Shongwe R Waligo A Mahdi M Mazia G Narayanan I Fuentes MER Strengthening postnatal care services including postpartum family planning in Kenya 2008 Washington, D.C.: Population council. Frontiers in reproductive health program (FRONTIERS)

[B22] Goldberg HI McNeil M Alison S Contraceptive use and fertility decline in chogoria, Kenya Stud Fam Plann 1989 20 1 17 25 10.2307/1966657 2711416

[B23] Solo J Billings DL Aloo-Obunga C Ominde A Makumi M Creating linkages between incomplete abortion treatment and family planning services in Kenya Stud Fam Plann 1999 30 1 17 27 10.1111/j.1728-4465.1999.00017.x 10216893

[B24] Lucas D Fertility and family planning in southern and central Africa Stud Fam Plann 1992 23 3 145 158 10.2307/1966724 1523695

[B25] Stekelenburg J Kyanamina S Mukelabai M Wolffers I van Roosmalen J Waiting too long: low use of maternal health services in Kalabo, Zambia Trop Med Int Health 2004 9 3 390 398 10.1111/j.1365-3156.2004.01202.x 14996369

[B26] MacKeith N Chinganya O Ahmed Y Murray S Zambian women's experiences of urban maternity care: results from a community survey in Lusaka Afr J Reprod Health/La Revue Africaine de la Santé Reproductive 2003 7 1 92 102 12828141

[B27] Ransjö-Arvidson A Christensson K Darkwah G Lungu F Kakoma C Chikamata D Diwan V Sterky G Maternity care routines in a teaching hospital in Zambia East Afr Med J 1989 66 7 427 2606024

[B28] Lagro M Liche A Mumba T Ntebeka R van Roosmalen J Postpartum care attendance at a rural district hospital in Zambia Trop Doct 2006 36 4 205 208 10.1258/004947506778604742 17034688

[B29] Lagro M Liche A Mumba T Ntebeka R van Roosmalen J Postpartum health among rural Zambian women Afr J Reprod Health 2003 7 3 41 48 10.2307/3583287 15055145

[B30] Nsemukila B Phiri D Diallo H Banda S Benaya WK Kitahara N A Study of Factors Associated with Maternal Mortality in Zambia 1998 Lusaka: Ministry of Health

[B31] Charurat E Bahir N Airede LR Abdu-Aguye S Otolorin E Mckaig C Postpartum Systematic Screening in Northern Nigeria: A Practical Application of Family Planning and Maternal Newborn and Child Health Integration 2010 Washington, DC: USAID and Access

[B32] Borda M Family Planning Needs during the First Year Postpartum. Unpublished 2006 Baltimore, MD: ACCESS-FP Project, JHPIEGO

